# Nano-curcumin improves glucose indices, lipids, inflammation, and Nesfatin in overweight and obese patients with non-alcoholic fatty liver disease (NAFLD): a double-blind randomized placebo-controlled clinical trial

**DOI:** 10.1186/s12986-019-0331-1

**Published:** 2019-01-28

**Authors:** Seyed Ali Jazayeri-Tehrani, Seyed Mahdi Rezayat, Siavash Mansouri, Mostafa Qorbani, Seyed Moayed Alavian, Milad Daneshi-Maskooni, Mohammad-Javad Hosseinzadeh-Attar

**Affiliations:** 1 Nutritional Sciences, Department of Clinical Nutrition, School of Nutritional Sciences and Dietetics, Tehran University of Medical Sciences, Tehran, Iran; 2 Department of Pharmacology, School of Medicine, Tehran University of Medical Sciences, Tehran, Iran; 3 Department of Medical Nanotechnology, School of Advanced Technologies in Medicine, Tehran University of Medical Sciences, Tehran, Iran; 4 Department of Toxicology–Pharmacology, Faculty of Pharmacy, Pharmaceutical Science Branch, Islamic Azad University (IAUPS), Tehran, Iran; 5 National Iranian Oil Company (NIOC) Central Hospital, Tehran, Iran; 6 Non-Communicable Diseases Research Center, Alborz University of Medical Sciences, Karaj, Iran; 7 Baqiyatallah Research Center for Gastroenterology and Liver Diseases (BRCGL), Baqiyatallah University of Medical Sciences, Tehran, Iran; 8 Nutritional Sciences, Department of Community Nutrition, School of Nutritional Sciences and Dietetics, Tehran University of Medical Sciences, Tehran, Iran; 9 Department of Clinical Nutrition, School of Nutritional Sciences and Dietetics, Tehran University of Medical Sciences, No.44, Hojjatdoust Alley, Naderi Ave, Keshavarz Blvd, Tehran, Iran; 10 School of Medicine, Jiroft University of Medical Sciences, Jiroft, Kerman, Iran

**Keywords:** Non-alcoholic fatty liver disease, Nano-curcumin, Obesity, Overweight, Iran

## Abstract

**Background:**

Since lifestyle changes are main therapies for non-alcoholic fatty liver disease (NAFLD), changing dietary components (nutritional or bioactive) may play a parallel important role. Few studies have assessed the effects of curcumin on NAFLD (mainly antioxidant and anti-inflammatory effects). We aimed to determine the effects of nano-curcumin (NC) on overweight/obese NAFLD patients by assessing glucose, lipids, inflammation, insulin resistance, and liver function indices, especially through nesfatin.

**Methods:**

This double-blind, randomized, placebo-controlled clinical trial was conducted in the Oil Company Central Hospital, Tehran. 84 overweight/obese patients with NAFLD diagnosed using ultrasonography were recruited according to the eligibility criteria (age 25–50 yrs., body mass index [BMI] 25–35 kg/m^2^). The patients were randomly divided into two equal NC (*n* = 42) and placebo (n = 42) groups. Interventions were two 40 mg capsules/day after meals for 3 months. Lifestyle changes were advised. A general questionnaire, a 24-h food recall (at the beginning, middle and end), and the short-form international physical activity questionnaire (at the beginning and end) were completed. Also, blood pressure, fatty liver degree, anthropometrics, fasting blood sugar (FBS) and insulin (FBI), glycated hemoglobin (HbA1c), homeostasis model assessment-insulin resistance (HOMA-IR), quantitative insulin sensitivity check index (QUICKI), total cholesterol (TC), triglyceride (TG), low-density lipoprotein cholesterol (LDL-c), high-density lipoprotein cholesterol (HDL-c), tumor necrosis factor-alpha (TNF-α), high sensitive c-reactive protein (hs-CRP), interleukin-6 (IL-6), liver transaminases, and nesfatin were determined at the beginning and end.

**Results:**

NC compared with placebo significantly increased HDL, QUICKI, and nesfatin and decreased fatty liver degree, liver transaminases, waist circumference (WC), FBS, FBI, HbA1c, TG, TC, LDL, HOMA-IR, TNF-α, hs-CRP, and IL-6 (*P* < 0.05). The mean changes in weight, BMI, body composition (BC), and blood pressure were not significant (*P* > 0.05). After adjustment for confounders, the changes were similar to the unadjusted model.

**Conclusion:**

NC supplementation in overweight/obese NAFLD patients improved glucose indices, lipids, inflammation, WC, nesfatin, liver transaminases, and fatty liver degree. Accordingly, the proposed mechanism for ameliorating NAFLD with NC was approved by the increased serum nesfatin and likely consequent improvements in inflammation, lipids, and glucose profile. Further trials of nano-curcumin’s effects are suggested.

**Trial registration:**

Iranian Registry of Clinical Trials, IRCT2016071915536N3. Registered 2016-08-02.

## Introduction

Non-alcoholic fatty liver disease (NAFLD) is the deposition of triglycerides (TG) in hepatocytes over than 5% of liver weight/volume and has three grades according to the liver biopsy (mild: < 33%, moderate: 33–66%, severe: > 66%). Usually, non-invasive diagnostic methods such as ultrasound, CT scan, and MRI are widely utilized that exact differentiation between the stages is difficult. Also, the liver enzymes (alanine transaminase [ALT] and aspartate transaminase [AST]) may be elevated 1.5–2 times above normal levels. However, many individuals with advanced non-alcoholic steatohepatitis (NASH) and even cirrhosis have normal liver enzyme levels. NAFLD rates are therefore likely higher than reported. Symptoms frequently include fatigue and upper-right quarter abdominal discomfort. The average adult prevalence is roughly 30% (65–85% and 15–20% in obese [BMI ≥ 25] and non-obese [BMI < 25] patients, respectively). It is more prevalent in males [1–4].

Two phases of pathology of NAFLD are fat deposition with hepatic steatosis and NASH. Insulin resistance plays a key role in both phases and common irritants include oxidative stress and inflammation. Liver fat content directly correlates with insulin resistance. Activating nuclear factor kappa B (NF-κB) upregulates pro-inflammatory cytokines, which can affect insulin activity. Inflammation, adipokines, oxidative stress or lipid metabolites can all, therefore, adjust insulin sensitivity, even though the intrahepatic fat content may not be necessarily directly related to these factors. The other risk factors include increased blood insulin levels, central obesity, type 2 diabetes, certain medications, nutrition status (starvation, protein or choline deficiency), some diseases, jejunum bypass, age, family history, malnutrition, severe weight loss, and gastrointestinal tract infections. Also, NAFLD incidence may correlate to the high saturated fat and/or carbohydrate intake. Other patients can display normal weight despite having abdominal obesity and insulin resistance [1–4].

Nesfatin as a neuropeptide of the hypothalamus is involved in appetite regulation and body fat storage by important functions in metabolizing glucose, phosphorylating specific signaling proteins, and increasing liver insulin sensitivity, specifically by AMP-activated protein kinase. Its gene is expressed in some locations including the brain, pancreas, stomach endocrine cells, and adipocytes. Nesfatin gene expression is activated by peroxisome proliferator-activated receptors (PPARs), especially PPARγ [5, 6]. Recently, serum nesfatin levels in overweight/obese NAFLD patients aged 30–60 years was significantly lower than in healthy individuals [1, 2, 6].

The common prescription for NAFLD involves lifestyle changes (weight loss and physical activity). Since weight loss and long-term maintenance is not always an easy task, the dietary modifications can be seen as a therapeutic approach for such patients. Accordingly, it is needed to investigate the effects of certain nutrients and/or dietary ingredients on NAFLD [1–4].

Curcumin as the effective ingredient in turmeric (ginger family) expresses multiple effects including antioxidant, anti-inflammatory, antimicrobial, and anti-carcinogenic ones. It raises both PPARγ activity and expression, which are important for inhibiting inflammation and oxidative stress as major factors involved in insulin resistance and NAFLD [1, 2].

The prevalence and implications of NAFLD are rising. Since there are no medications to combat it and the role of nutrition is a key treatment factor; by examining dietary components such as curcumin for NAFLD improvement, researchers can begin to uncover new treatments. Curcumin is involved in a number of metabolic functions in improving insulin resistance. Despite multiple health benefits, its low stability and bioavailability impact its therapeutic efficiency. Various techniques have been explored recently to improve this problem by using of polymeric nanoparticles called Nano-curcumin (NC) (for example, polylactic-co-glycolic acid (PLGA) nanoparticles can increase curcumin bioavailability 22-fold) [1, 2]. In this trial, NC was used, therefore. The onset and progression of NAFLD may be improved by curcumin through inhibiting inflammation and oxidative stress. The severity of NAFLD is increased by overweight or obesity; however, no human studies have been carried out to determine how these are affected by curcumin. This study, therefore, was designed to examine effects of curcumin on blood glucose indices, lipids, inflammatory profiles, liver function (fatty liver degree, ALT, AST), and insulin resistance (HOMA-IR, QUICKI), especially by looking at serum nesfatin in obese NAFLD patients.

## Materials and methods

### study design and participants

The ethics committee of Tehran University of Medical Sciences approved this double-blind randomized placebo-controlled clinical trial as IR.TUMS.REC.1394.791. The study was registered to the Iranian Registry of Clinical Trials as IRCT2016071915536N3 on 02/08/2016. The participants were overweight or obese NAFLD patients referred to the sonography department of the NIOC Central Hospital in Tehran.

**Inclusion criteria** included NAFLD according to ultrasonography, aged 25–50, and 25 ≤ BMI < 35 kg/m^2^. **Exclusion criteria** included alcohol intake during the previous year, inability/unwillingness to cooperate, other liver conditions, secondary NAFLD, uncontrolled hypertension (> 140/90 mmHg), pregnancy or lactation, professional athlete status, use of statins, ursodeoxycholic acid, probiotics, antihypertensive, curcumin-interactive drugs, consumption of multivitamin/mineral and antioxidant supplements over the previous 3 months, weight loss over the previous 3 months, and not taking more than 10% of the intervention supplement [1–4, 7–9].

### Randomization and intervention

**Fig. 1:**
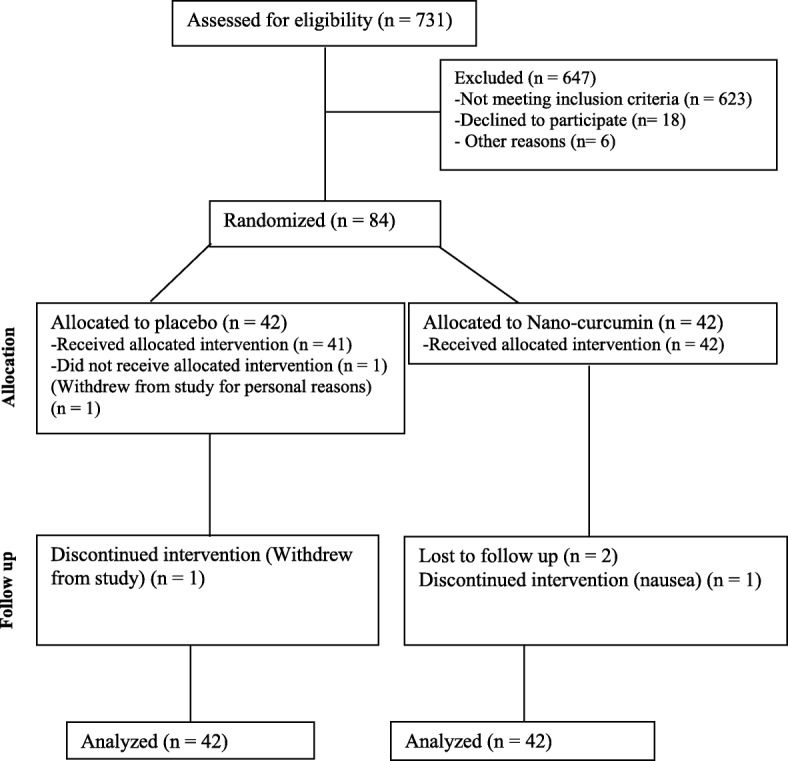
Flowchart of participants with overweight/obesity and NAFLD

Subjects were divided into two equal groups by the block randomization method, carried out by an assistant (NC [*n* = 42] or placebo [n = 42]). Stratified randomization was used to control for sex. The ratio of the two groups was 1:1. 3 patients from the NC group and 2 patients from the placebo group declined to participate (Fig. 1).

Intervention allocation blinding was performed for both participants and investigators before the beginning, kept during the intervention, and opened after the data analysis by a field worker. The NC and placebo capsules were supplied by the *Exir-Nano-Sina* Company. The capsules were similar in shape, size, and color. The *sinacurcumin*® dose was 80 mg/day (two 40-mg capsules per day, according to the manufacturer’s instructions: one capsule at breakfast and another at dinner). The supplements were distributed monthly, and compliance status was assessed monthly by face-to-face consultation, and weekly by phone. The compliance percentage of supplements was calculated according to the mean percentage of the number of capsules consumed by the individuals of any groups. The lifestyle advice was equally presented by a trained dietician (SAJT) at the hospital.

### Assessments and measurements

#### General characteristics, dietary intakes, and physical activity

The main investigator identified NAFLD patients, checked their eligibility criteria, clarified the study details, and obtained informed consent. Interviews were conducted to fill the questionnaires, including a general questionnaire, a 24-h food recall (at beginning, middle, and end), and the short-form IPAQ (SF-IPAQ) (at beginning and end). At the commencement of the trial, lifestyle advice [10] was presented by a qualified dietician, including adhering to a low-calorie diet aimed at achieving weight loss of about 0.5–1 kg/week, and increasing physical activity by undertaking moderate-intensity aerobic exercise at least 3 times/week for 30–45 min [11].

Subjects’ dietary status was determined by *Nutritionist-4* software, using gram/day values from the 24-h food recall (validated in Iran [12]) [12, 13].

The IPAQ short-form questionnaire has 7 stratified questions that determine one of three overall activity levels. This questionnaire has previously been validated in Iran [14, 15].

#### Anthropometric measurements

Weight (at beginning and end) and height (at the beginning) were determined using a digital scale and stadiometer (*Seca® Germany, Model: 7551021994*). BMI was calculated by dividing weight in kilograms by height in meters squared. Body composition percentages, including body fat and lean body mass, were measured using the *Bioimpedance Analyzer* device (Tanita®). At the beginning and end of the study, systolic and diastolic blood pressures were obtained with the use of a mercury manometer (Riester®) and were reported in mmHg. Waist circumference was measured at the middle of the last rib and at the iliac crest with minimal clothing, using a non-elastic tape, at the beginning and end of the trial. Blood sampling, storage, and laboratory tests were carried out at the NIOC Central Hospital, Tehran.

#### Sonography and blood biomarkers measurements

The ultrasound test was performed by a radiologist, using a sonography device (*General Electric (GE)®*, *Model: Voluson E8*) after a 12-h fast, to reduce potential human error.

10 ml of blood (at the beginning and end) was taken from the peripheral vein following a 12-h overnight fast and was centrifuged for 20 min (3000 *g*). Serum glucose indices, lipids, and liver transaminases were assessed on the day of the blood sample. The remaining serum was frozen and stored at − 80 °C before analysis.

Serum nesfatin, IL-6, and TNF-α were measured using sandwich ELISA and the following kits: *Shanghai Crystal Day Biotech Co. Ltd®; Intra-assay CV < 8%, Inter-assay CV < 10%* with an automatic device (*Elisys Uno Human®*). The ELISA kit for FBI and hs-CRP were *DiaMetra® Co. of Italy, DCM076–8; Intra-assay CV ≤ 5%, Inter-assay CV ≤ 10%* and *Diagnostics Biochem Canada (DBC) Inc®, REF: CAN-CRP-4360, Version 5.0; Intra-assay CV ≤ 15.2%, Inter-assay CV ≤ 9.9%*, respectively. Serum glucose was determined with the glucose oxidase method, using a *Hitachi analyzer* device (*q17®*) and the specified kit *Bionik®, Liquid Stable, Glucose oxidase GOD-POD, Mono-reagent; Intra-assay CV ≤ 2.10%, Inter-assay CV ≤ 3.09%*. HOMA-IR and QUICKI indexes were calculated by the following formulas:

$$ QUICKI= 1/\left(\mathit{\log}\  fasting\ insulin\ \left[\mu IU/ ml\right]\right)+\mathit{\log}\  fasting glucose\ \left[ mg/ dl\right]\Big). $$

*HOMA* ‐ *IR* = *FBI* [*μIU*/*ml*] × *FBS* [*mg*/*dl*]/*405*.

Serum lipids (TC, TG, HDL-c, LDL-c), ALT, AST, and HbA1c were assessed with the *Hitachi analyzer* device (*q17®*) and specific kits (Table 1).

**Table 1:** The used kits for determination of the serum lipids, liver enzymes, and glycosylated hemoglobin

Factors	Kits
TC	*Bionik®, Liquid Stable, Enzymatic Colorimetric CHOD-POD, Intra-assay CV ≤ 1.216%, Inter-assay CV ≤ 6.906%*
HDL	*Bionik®, Liquid Stable, Direct. Enzymatic Colorimetric, Intra-assay CV ≤ 0.7%, Inter-assay CV ≤ 1.5%*
TG	*Bionik®, Liquid Stable, GPO-POD, Mono-reagent, Intra-assay CV ≤ 1.573%, Inter-assay CV ≤ 7.704%*
LDL	*Bionik®, Liquid Stable, Direct. Enzymatic Colorimetric, Intra-assay CV ≤ 1.76%, Inter-assay CV ≤ 0.65%*
AST	*Bionik®, Liquid Stable, NADH. Kinetic UV.Liquid, Intra-assay CV ≤ 3.02%, Inter-assay CV ≤ 3.00%*
ALT	*Bionik®, Liquid Stable, NADH. Kinetic UV.IFCC, Intra-assay CV ≤ 4.27%, Inter-assay CV ≤ 4.68%*
HbA1c	*Bionik®, Liquid Reagents, Particle Enhanced Immuno-Assay [PEIA], Intra-assay CV ≤ 1.72%, Inter-assay CV ≤ 2.77%*

### Sample size

According to Chuengsamarn et al. [16], the mean ± SD of HOMA-IR indices in the curcumin and placebo groups were 3.22 ± 1.30 and 4.08 ± 1.35, respectively. There were 42 participants in each sample group, with a CI of 95%, power of 80%, and a drop of 15%. 84 individuals in total were chosen and split into two equal groups (NC and placebo) using a block randomization method.

### Data analysis and accessibility

Data management was performed, including entry, security, coding, and storage. The follow-up data missing from patients was estimated by the modified-intention to treat (m-ITT) analysis, and the regression imputation method. The Kolmogorov-Smirnov, Chi-square, Fisher Exact, and t or Mann-Whitney tests were implemented to assess the normality of continuous variables and categorical and continuous baseline characteristics, respectively. Two-way repeated measures analysis of variance (TWRM-ANOVA) was used to compute time effects and time by treatment interaction effects with all the dependent variables. Moreover, TWRM-ANOVA was adjusted for participants’ dietary intake of energy, total fat, saturated fat, monounsaturated fatty acid, vitamins D, B1, B6, and folate. A 95% confidence interval (CI) and a *P*-value of < 0.05 were used to report data. Data analysis was performed using SPSS_16_ (statistical package for the social sciences) and STATA_11SE_ (general-purpose statistical software package by Stata Corp) software. The main researcher had full access to the finalized dataset and presented the results.

## Results

### Participant traits

As shown in Figs. 1, 731 people were screened based on their medical history. 108 subjects met the eligibility criteria, of whom 18 declined to take part, and 6 could not participate. 84 subjects were randomized into 2 groups and completed the first visit. At the follow-up stage, 5 subjects could not continue (for personal reasons and/or travel; NC *n* = 3; placebo *n* = 2). Eventually, data analysis was carried out on the 84 subjects, according to the modified-ITT analysis.

Participant general traits and physical activity levels are displayed in Table 2. The majority of the subjects had similar education levels and presented the high economic status and low physical activity scores. Both groups well consumed the prescribed supplements. The compliance percentage of supplements were 95.98% in NC group and 97.95% in placebo group.

**Table 2:** General traits and physical activity levels of overweight/obese patients with non-alcoholic fatty liver disease (NAFLD)

Variables			Nanocurcumin (*n* = 42) n(%) or Mean(SD)	Placebo (n = 42) n(%) or Mean(SD)	*P*-value
Age (yrs)			41.8(5.6)	42.5(6.2)	0.2^*^
Gender	male		23(54.8)	23(54.8)	1.0^**^
	female		19(45.2)	19(45.2)	
Height (cm)			167.8(9.8)	167.7(9.0)	0.8^*^
Marriage status	single		5(11.9)	7(16.7)	0.5^**^
	married		37(88.1)	35(83.3)	
Job status	employee, free job/retired		31(73.8)	25(59.5)	0.1^**^
	housewife, unemployed		11(26.2)	17(40.5)	
Education level	up to associate degree		21(50)	20(47.6)	0.8^**^
	Bachelor and higher		21(50)	22(52.4)	
Economic level	Low (≤3 living items)		0(0)	0(0)	0.8^**^
	moderate (4–6 living items)		19(45.2)	18(42.9)	
	High (≥7 living items)		23(54.8)	24(57.1)	
Physical activity level (Baseline)	low (< 600 MET-minutes/week)		31(73.8)	28(66.7)	0.4^**^
	Moderate (600 to < 1500 MET-minutes/week)		11(26.2)	14(33.3)	
	High (≥ 1500 MET-minutes/week)		0(0)	0(0)	
Physical activity level (After 3 months)	low (< 600 MET-minutes/week)		27(64.3)	26(61.9)	0.8^$^
	Moderate (600 to < 1500 MET-minutes/week)		15(35.7)	16(38.1)	
	High (≥ 1500 MET-minutes/week)		0(0)	0(0)	
Fatty liver (Baseline)	No		0(0)	0(0)	0.5^**^
	Yes	Mild	35(83.3)	37(88.1)	
		Moderate	7(16.7)	5(11.9)	
		Severe	0(0)	0(0)	
Fatty liver (After 3 months)	No		20(47.6)	5(11.9)	< 0.001^**^
	Yes	Mild	21(50.0)	33(78.6)	
		Moderate	1(2.4)	4(9.5)	
		Severe	0(0)	0(0)	

*Mann-Whitney; **Chi-square; **$**Fisher exact test

Dietary vitamin D intake at baseline was greater in the placebo group, while other baseline features between the two groups were similar (Tables 2, 3, 4).

**Table 3:** Comparison of baseline mean for weight, BMI, glucose indices, nesfatin, inflammatory factors, and liver enzymes in overweight/obese patients with non-alcoholic fatty liver disease (NAFLD)

Variables	Nanocurcumin (n = 42) Mean(SD)	Placebo (n = 42) Mean(SD)	*P*-value
Weight (kg)	86.54(10.98)	86.70(11.15)	0.9^*^
BMI (kg/m2)	30.67(2.14)	30.75(2.35)	0.9^**^
Waist circumference (cm)	105.4(6.2)	103.8(6.7)	0.8^*^
Fat Mass (%)	31.6(6.4)	31.9(4.7)	0.8^*^
SBP (mmhg)	120.3(4.7)	120.7(4.3)	0.7^**^
DBP (mmhg)	78.8(4.5)	79.8(4.3)	0.1^*^
ALT (u/l)	42.8(11.6)	42.1(8.2)	0.7^**^
AST (u/l)	28.4(6.7)	27.6(7.8)	0.4^**^
TC (mg/dl)	212.9(18.9)	211.8(21.4)	0.8^*^
LDL-c (mg/dl)	135.6(17.6)	133.0(20.7)	0.6^*^
TG (mg/dl)	175.9(70.3)	181.2(65.6)	0.7^**^
HDL-c (mg/dl)	41.8(5.6)	42.7(5.7)	0.4^*^
FBS (mg/dl)	89.1(5.4)	89.3(5.8)	0.9^*^
HbA1c (%)	5.2(0.2)	5.3(0.2)	0.4^**^
Nesfatin (ng/ml)	1.8(0.5)	1.8(0.4)	0.5^*^
FBI (μIU/ml)	8.0(0.5)	7.9(0.6)	0.7^*^
TNF-α (ng/l)	14.7(3.3)	15.1(2.7)	0.2^*^
IL-6 (ng/l)	7.6(1.5)	7.9(1.5)	0.2^*^
hs-CRP (mg/l)	5.9(2.5)	5.3(2.5)	0.2^**^
HOMA-IR (score)	1.77(0.16)	1.75(0.17)	0.7^**^
QUICKI (score)	0.3505(0.0049)	0.3509(0.0057)	0.7^*^

*t-test; **Mann-Whitney; *ALT* alanine transaminase, *AST* aspartate transaminase, *BMI* body mass index, *FBI* fasting blood insulin,*FBS* fasting blood sugar, *HOMA-IR* homeostasis model assessment-insulin resistance, *hs-CRP* high-sensitive C-reactive protein, *HDL-C* high density lipoprotein-cholesterol, *IL-6* interleukin-6, *LDL-C* low density lipoprotein-cholesterol, *QUICKI* quantitative insulin sensitivity check index, *TC* total cholesterol, *TNF-α* tumor necrosis factor-alpha

**Table 4:** Dietary intakes of overweight/obese patients with non-alcoholic fatty liver disease (NAFLD)

Dietary intakes		Nanocurcumin (*n* = 42) Mean(SD)	Placebo (n = 42) Mean(SD)	*P*-value	
Energy (kcal)	Baseline	2473.2(470.5)	2338.6(496.3)	0.2^*^	0.001^**^
	1.5 Months	2089.9(412.9)	2267.0(468.4)	0.07	
	3 Months	2019.4(380.5)	2205.9(476.0)	0.05	
Protein (g)	Baseline	100.7(30.7)	97.1(32.7)	0.6	0.3
	1.5 Months	93.7(24.9)	93.8(31.8)	0.8	
	3 Months	84.1(28.7)	92.9(31.4)	0.2	
Protein (%)	Baseline	16.3(3.7)	16.5(4.1)	0.7	0.2
	1.5 Months	18.0(3.8)	16.4(3.9)	0.06	
	3 Months	16.6(4.8)	16.7(4.1)	0.8	
Carbohydrate (g)	Baseline	296.1(55.6)	280.5(81.9)	0.3	0.3
	1.5 Months	253.8(68.9)	252.3(58.9)	0.9	
	3 Months	256.2(47.8)	266.0(73.3)	0.3	
Carbohydrate (%)	Baseline	48.5(7.7) %	47.8(9.0) %	0.7	0.5
	1.5 Months	48.4(8.7) %	45.1(8.7) %	0.08	
	3 Months	51.2(7.4) %	48.1(8.0) %	0.07	
Fat total (g)	Baseline	103.1(32.8)	97.0(27.4)	0.3	0.001
	1.5 Months	82.3(22.5)	102.4(31.1)	0.001	
	3 Months	78.7(20.6)	90.4(24.6)	0.02	
Fat total (%)	Baseline	37.00(6.9) %	37.5(7.4) %	0.7	0.1
	1.5 Months	35.5(6.7) %	40.2(7.1) %	0.003	
	3 Months	34.8(4.9) %	37.0(6.6) %	0.09	
Cholesterol (mg)	Baseline	268.2(168.8)	282.3(178.7)	0.7	0.3
	1.5 Months	273.9(150.8)	271.3(153.1)	0.9	
	3 Months	198.3(126.8)	263.9(125.6)	0.01	
Saturated fat (g)	Baseline	27.3(11.4)	25.5(9.2)	0.7	0.03
	1.5 Months	22.5(7.2)	27.9(9.7)	0.005	
	3 Months	21.2(8.5)	25.6(9.8)	0.03	
Monounsaturated fatty acid (g)	Baseline	37.8(13.7)	36.1(9.8)	0.5	0.008
	1.5 Months	30.4(8.9)	36.7(11.6)	0.006	
	3 Months	28.5(8.2)	33.7(9.0)	0.008	
Polyunsaturated fatty acid (g)	Baseline	26.4(13.6)	25.0(11.6)	0.5	0.06
	1.5 Months	20.3(7.6)	26.6(15.3)	0.1	
	3 Months	20.2(6.8)	22.1(7.1)	0.4	
Vitamin A (RAE) (μg)	Baseline	316.8(210.7)	349.0(264.2)	0.6	0.6
	1.5 Months	366.9(287.1)	319.7(270.6)	0.2	
	3 Months	416.2(378.3)	407.0(344.8)	0.4	
Carotenoids (μg)	Baseline	8009.3(6994.8)	7580.6(6601.1)	0.7	0.8
	1.5 Months	8527.0(6850.7)	8068.6(6627.9)	0.7	
	3 Months	8184.9(6718.9)	8705.7(7724.2)	0.9	
Vitamin C (mg)	Baseline	97.0(84.4)	79.8(61.3)	0.4	0.9
	1.5 Months	83.8(61.0)	74.2(69.4)	0.1	
	3 Months	95.1(73.5)	85.7(71.8)	0.2	
Calcium (mg)	Baseline	1086.0(502.2)	1115.7(521.2)	0.7	0.1
	1.5 Months	1025.8(360.1)	910.2(473.1)	0.03	
	3 Months	897.8(408.9)	1076.8(475.1)	0.06	
Iron (mg)	Baseline	15.0(3.5)	14.8(5.0)	0.7	0.3
	1.5 Months	14.1(4.0)	13.7(3.1)	0.6	
	3 Months	13.1(3.1)	14.1(4.0)	0.1	
Vitamin D (μg)	Baseline	1.0(1.8)	1.9(2.1)	0.01	0.02
	1.5 Months	2.4(3.6)	1.1(1.6)	0.03	
	3 Months	2.1(3.5)	1.4(1.6)	0.9	
Vitamin E (mg)	Baseline	29.7(19.0)	28.7(13.1)	0.7	0.5
	1.5 Months	24.9(9.2)	27.0(14.6)	0.4	
	3 Months	23.7(8.6)	26.9(8.2)	0.1	
Vitamin B1 (mg)	Baseline	1.9(0.3)	1.7(0.5)	0.08	0.01
	1.5 Months	1.6(0.4)	1.5(0.4)	0.3	
	3 Months	1.5(0.3)	1.7(0.5)	0.06	
Vitamin B2 (mg)	Baseline	1.9(0.7)	2.0(0.8)	0.5	0.1
	1.5 Months	1.9(0.6)	1.8(0.6)	0.1	
	3 Months	1.7(0.7)	1.9(0.7)	0.1	
Vitamin B3 (mg)	Baseline	30.3(13.2)	27.8(12.9)	0.4	0.4
	1.5 Months	25.7(10.2)	27.2(14.7)	0.8	
	3 Months	24.2(10.6)	26.3(11.9)	0.4	
Vitamin B6 (mg)	Baseline	2.2(0.7)	1.9(0.6)	0.05	0.02
	1.5 Months	1.8(0.5)	1.8(0.7)	0.5	
	3 Months	1.7(0.6)	1.9(0.6)	0.1	
Folate (DFE) (μg)	Baseline	504.0(222.7)	443.6(181.5)	0.3	0.04
	1.5 Months	425.7(155.8)	426.5(149.7)	0.7	
	3 Months	413.0(126.7)	471.7(152.9)	0.05	
Vitamin B12 (μg)	Baseline	4.1(2.5)	4.4(2.5)	0.6	0.6
	1.5 Months	5.1(2.8)	4.6(2.5)	0.5	
	3 Months	4.3(3.0)	4.4(2.4)	0.9	
Vitamin K (μg)	Baseline	148.6(224.4)	134.9(287.3)	0.1	0.8
	1.5 Months	142.6(279.7)	86.5(135.3)	0.5	
	3 Months	192.2(366.3)	168.8(419.1)	0.6	
Zinc (mg)	Baseline	12.4(3.3)	12.7(3.7)	0.7	0.7
	1.5 Months	12.6(4.0)	12.4(3.3)	0.7	
	3 Months	11.5(3.7)	12.1(4.5)	0.6	
Selenium (μg)	Baseline	113.7(34.2)	119.9(54.1)	0.7	0.2
	1.5 Months	120.4(58.1)	99.2(37.9)	0.09	
	3 Months	112.0(57.7)	106.6(44.5)	0.6	
Fiber total (g)	Baseline	29.8(12.8)	31.0(16.1)	0.9	0.09
	1.5 Months	30.1(16.5)	27.1(15.1)	0.5	
	3 Months	24.5(9.5)	31.5(15.6)	0.03	

*Total of the column: t-test or Mann-Whitney; **Total of the column: Two way repeated measures-ANOVA (TWRM-ANOVA)

### Dietary status and measured biomarkers changes

The dietary intakes of energy, total fat, saturated fat, monounsaturated fatty acid, vitamins D, B1, B6, and folate during the study were higher in the placebo group (*P* < 0.05, Table 4), while other dietary patterns were almost identical between groups. These significant differences in terms of consumption were later considered as confounders in the final analysis model.

In the NC group, the mean difference of DBP was not significant (*P* > 0.05), although weight, BMI, WC, SBP, ALT, AST, TC, LDL-c, TG, FBS, FBI, HbA1c, FM, TNF-α, IL-6, hs-CRP, and HOMA-IR decreased, and HDL-c, QUICKI, and Nesfatin increased significantly (*P* < 0.05). In the placebo group, the mean difference of SBP was not significant (*P* > 0.05), but the weight, BMI, WC, DBP, ALT, AST, TC, LDL-c, TG, FBS, FBI, HbA1c, FM, TNF-α, IL-6, hs-CRP, and HOMA-IR decreased, and HDL-c, QUICKI, and Nesfatin increased significantly (*P* < 0.05).

The mean ± standard deviation of BMI at baseline and at the end of intervention were 30.6 ± 2.14 kg/m^2^ and 29.7 ± 2.10 kg/m^2^ in NC group, and 30.7 ± 2.35 kg/m^2^ and 29.9 ± 2.53 kg/m^2^ in the placebo group. These mean changes remained non-significant in both the unadjusted (*P* = 0.2) and adjusted models (*P* = 0.3).

As can be seen in the time by treatment interaction effect in both the unadjusted and adjusted analysis models, WC, ALT, AST, TC, LDL-c, TG, FBS, FBI, HbA1c, TNF-α, IL-6, hs-CRP, and HOMA-IR decreased, and HDL-c, QUICKI, and Nesfatin increased significantly among the NC group in comparison with the placebo group (*P* < 0.05) (Table 5). In other words, NC, in comparison with placebo, significantly increased HDL-c, QUICKI, and Nesfatin and decreased WC, ALT, AST, TC, LDL-c, TG, FBS, FBI, HbA1c, TNF-α, IL-6, hs-CRP, and HOMA-IR (*P* < 0.05). After adjustment for confounders, the significant changes remained similar (*P* < 0.05).

**Table 5:** The changes in weight, BMI, glucose indices, nesfatin, inflammatory factors, and liver enzymes in overweight/obese NAFLD patients

Variables	Intervention	Baseline Mean(SD)	3 Months Mean(SD)	*P*-value^$^	Mean Difference (95% CI)	*P*-value^#^		
						Time	Treatment	Interaction
Weight (kg)	Nanocurcumin (n = 42)	86.5 (10.9)	83.7 (10.7)	< 0.001	−2.8 (−3.5, −2.0)	< 0.001	0.8	0.3
	Placebo (n = 42)	86.7(11.0)	84.3(11.0)	< 0.001	−2.4 (−3.1, −1.6)	< 0. 1	0.3	0.3
BMI (kg/m^2^)^*^	Nanocurcumin (*n* = 42)	30.6(2.14)	29.7(2.10)	< 0.001	−0.9 (− 1.0, −0.7)	< 0.001	0.6	0.2
	Placebo (n = 42)	30.7(2.35)	29.9(2.53)	< 0.001	− 0.8 (− 0.9, − 0.6)	0.03	0.9	0.3
Fat Mass (%)	Nanocurcumin (*n* = 42)	31.6(6.4)	30.3(6.5)	< 0.001	−1.3 (− 1.7, − 0.8)	< 0.001	0.7	0.3
	Placebo (*n* = 42)	31.9(4.7)	30.7(4.9)	< 0.001	−1.2 (− 1.5, − 0.8)	0.003	0.1	0.9
Waist circumference (cm)	Nanocurcumin (n = 42)	105.4(6.2)	99.6(5.7)	< 0.001	−5.8 (−6.2, −5.3)	< 0.001	0.6	< 0.001
	Placebo (n = 42)	103.8(6.7)	102.5(6.9)	< 0.001	−1.3 (− 1.7, − 0.8)	0.07	0.9	< 0.001
SBP (mmhg)	Nanocurcumin (n = 42)	120.3(4.7)	118.2(4.4)	0.008	−2.1 (−2.4, −1.7)	0.001	0.3	0.3
	Placebo (n = 42)	120.7(4.3)	119.6(4.9)	0.07	−1.1 (−1.4, −0.7)	0.7	0.4	0.8
DBP (mmhg)	Nanocurcumin (n = 42)	78.8(4.5)	77.9(2.9)	0.102	−0.9 (−1.1, − 0.6)	0.008	0.2	0.7
	Placebo (n = 42)	79.8(4.3)	78.7(3.4)	0.03	−1.1 (−1.3, 0.8)	0.4	0.4	0.7
ALT (u/l)^**^	Nanocurcumin (n = 42)	42.8(11.6)	32.6(9.9)	< 0.001	−10.2 (− 10.9, −9.4)	< 0.001	0.1	< 0.001
	Placebo (n = 42)	42.1(8.2)	39.6(7.5)	0.001	−2.5 (−3.0, 1.9)	0.4	0.3	< 0.001
AST (u/l)^	Nanocurcumin (n = 42)	28.43(6.7)	22.03(5.9)	< 0.001	−6.4 (−6.8, −5.9)	< 0.001	0.3	< 0.001
	Placebo (n = 42)	27.60(7.8)	25.63(7.2)	0.002	−1.97 (−2.47, − 1.46)	0.052	0.7	< 0.001
TC (mg/dl)	Nanocurcumin (n = 42)	212.9(18.9)	195.2(19.8)	< 0.001	−17.7 (− 19.0, − 16.3)	< 0.001	0.2	0.002
	Placebo (n = 42)	211.8(21.8)	205.0(20.5)	0.005	−6.8 (−8.2, 5.3)	0.3	0.9	0.01
LDL-c (mg/dl)	Nanocurcumin (n = 42)	135.6(17.6)	114.6(20.5)	< 0.001	−21.0 (−22.2, −19.7)	< 0.001	0.3	< 0.001
	Placebo (n = 42)	133.0(20.7)	125.7(22.2)	< 0.001	−7.3 (−8.7, −5.8)	0.01	0.9	0.003
TG (mg/dl)	Nanocurcumin (n = 42)	175.9(70.3)	142.5(49.9)	< 0.001	−33.4 (−37.5, −29.2)	< 0.001	0.1	< 0.001
	Placebo (n = 42)	181.2(65.6)	175.3(62.5)	0.007	−5.9 (−10.2, −1.5)	0.5	0.057	0.001
HDL-c (mg/dl)	Nanocurcumin (n = 42)	41.8(5.6)	51.4(6.6)	< 0.001	9.6 (9.1, 10.0)	< 0.001	0.009	< 0.001
	Placebo (n = 42)	42.7(5.7)	43.8(5.4)	0.001	1.1 (0.7, 1.4)	< 0.001	0.04	< 0.001
FBS (mg/dl)	Nanocurcumin (n = 42)	89.1(5.4)	86.3(5.2)	< 0.001	−2.8 (−3.1, −2.4)	< 0.001	0.3	< 0.001
	Placebo (n = 42)	89.3(5.8)	88.2(5.5)	< 0.001	−1.1 (−1.4, 0.7)	0.6	0.2	0.03
HbA1c (%)	Nanocurcumin (n = 42)	5.2(0.218)	5.1(0.229)	< 0.001	−0.1 (−0.115, −0.085)	< 0.001	0.1	0.001
	Placebo (n = 42)	5.3(0.218)	5.2(0.188)	< 0.001	−0.1 (− 0.114, − 0.086)	0.01	0.8	0.002
Nesfatin (ng/ml)	Nanocurcumin (n = 42)	1.81(0.54)	3.37(8.8)	< 0.001	1.56 (1.52, 1.59)	< 0.001	< 0.001	< 0.001
	Placebo (n = 42)	1.88(0.49)	2.06(7.6)	< 0.001	.18 (0.14, 0.21)	< 0.001	0.003	< 0.001
FBI (μIU/ml)	Nanocurcumin (n = 42)	8.0(0.5)	6.5(0.9)	< 0.001	−1.5 (−1.54, −1.45)	< 0.001	0.001	< 0.001
	Placebo (n = 42)	7.9(0.6)	7.6(0.7)	< 0.001	−0.3 (−0.34, −0.25)	0.3	0.08	< 0.001
TNF-α (ng/l)	Nanocurcumin (n = 42)	14.7(3.3)	7.3(2.9)	< 0.001	−7.4 (−7.5, −7.2)	< 0.001	< 0.001	< 0.001
	Placebo (n = 42)	15.1(2.7)	13.7(4.0)	< 0.001	1.4 (−1.63, −1.16)	0.002	< 0.001	< 0.001
IL-6 (ng/l)	Nanocurcumin (n = 42)	7.60(1.56)	3.81(1.63)	< 0.001	−3.79 (−3.88, −3.69)	< 0.001	< 0.001	< 0.001
	Placebo (n = 42)	7.99(1.52)	7.02(7.6)	< 0.001	−0.97 (−1.07, −0.86)	< 0.001	< 0.001	< 0.001
hs-CRP (mg/l)	Nanocurcumin (n = 42)	5.9(2.57)	3.6(1.58)	< 0.001	−2.3 (−2.44, −2.15)	< 0.001	0.3	< 0.001
	Placebo (n = 42)	5.3(2.50)	5.2(2.47)	< 0.001	0.1 (−0.26, 0.06)	< 0.001	0.3	< 0.001
HOMA-IR (score)	Nanocurcumin (n = 42)	1.77(0.16)	1.39(0.21)	< 0.001	−0.38 (− 0.39, − 0.36)	< 0.001	0.001	< 0.001
	Placebo (n = 42)	1.75(0.17)	1.65(0.18)	< 0.001	0.1 (−0.11, − 0.08)	0.3	0.04	< 0.001
QUICKI (score)	Nanocurcumin (n = 42)	0.3505(0.0049)	0.3643(0.0092)	< 0.001	0.0138 0.0143, 0.0133)	< 0.001	0.001	< 0.001
	Placebo (n = 42)	0.3509(0.0057)	0.3543(0.0068)	< 0.001	0.0034 (0.0038, 0.0029)	0.3	0.3	< 0.001

*Inversely transformed; **Transformed by square root; **^**Logarithmically transformed; **$**Paired t-test; **#**Two way repeated measures-ANOVA (TWRM-ANOVA), top row: unadjusted; bottom row: adjusted for energy, total fat, saturated fat, monounsaturated fatty acid, vitamins D, B1, B6, and folate. *ALT* alanine transaminase, *AST* aspartate transaminase, *BMI* body mass index, *FBI* fasting blood insulin,*FBS* fasting blood sugar, *HOMA-IR* homeostasis model assessment-insulin resistance, *hs-CRP*: high-sensitive C-reactive protein, *HDL-C*: high density lipoprotein-cholesterol, *IL-6*: interleukin-6, *LDL-C*: low density lipoprotein-cholesterol, *QUICKI*: quantitative insulin sensitivity check index, *TC*: total cholesterol, *TNF-α*: tumor necrosis factor-alpha

### Safety

The patients reported no side-effects and side-events associated with treatment during the study, with the exception of one patient in the NC group, who reported nausea.

## Discussion

This trial was the first to assess the effects of NC on serum levels of some important factors related to overweight, obesity, and NAFLD.

The baseline variables were similar between the two groups with an exception for the dietary vitamin D intake was higher in the placebo group. Possible reasons for high entry similarity include single center selection and similar participant socio-economic levels.

According to both the unadjusted and adjusted analysis, NC (in comparison with placebo) significantly increased HDL-c, QUICKI, and nesfatin, and decreased WC, ALT, AST, TC, LDL-c, TG, FBS, FBI, HbA1c, TNF-α, IL-6, hs-CRP, and HOMA-IR. The changes in weight, BMI, and FM among the NC group in comparison with placebo were not significant.

Many studies have shown the significant effects of curcumin (especially more accessible forms such as NC) on anthropometric measurements (weight, BMI, WC, FM) [6, 17–25]. The different mechanisms have been proposed for effects of curcumin on anthropometric measurements including inhibition of the adipocytes, lipogenesis, fat mass, and inflammation, increasing lipolysis, energy consumption [26, 27], and brown fat tissue, and probiotics-like effects [24]. Also, the effects of nesfatin on anthropometrics include decreased appetite [28, 29], BMI [26, 30, 31], and WC [26]. Therefore, the WC improvement in this study may be attributed to the increased levels of serum nesfatin by NC supplementation.

In a study on NAFLD, curcumin not changed SBP and DBP significantly [17], although, SBP was significantly decreased in the NC group in this study. However, the beneficial effects of curcumin on blood pressure have been reported in two reviews [20, 27]. Some mechanisms have been proposed including improvement of vascular reactivity [27], inflammation, oxidative stress, and anthropometrics [20]. The studies of nesfatin’s effects on hypertension are divisive [32]. The reason for no changes of BP may be the normal BP of the participants at baseline.

The turmeric in a study on NAFLD patients significantly reduced, HOMA-R, FBS, and FBI [22]. Several reviews have reported the beneficial effects of curcumin on the glucose indices (FBS, HbA1c) [23, 24, 33, 34]. Some mechanisms were decreasing inflammatory factors, glucose production, glycogenolysis, insulin resistance, adipocytes, FM, weight and increasing antioxidant activity, glucose uptake, catabolism, and insulin sensitivity [24, 33, 35, 36]. In a study among diabetics, curcumin significantly reduced TC, non-HDL-c, and Lp(a) and increased HDL-c. TG and LDL-c significantly decreased only in the curcumin group [37]. The reasons for some mismatches may be the differences among patients and the supplement type and dose. In two separate studies in diabetic [18] and dyslipidemic patients [38], curcumin significantly reduced TG but no other lipids. The reasons of difference may be the different patients, intervention duration, and supplementation type and dose. The beneficial effects of curcumin on lipids have been reported in several reviews [18, 24, 33–53]. The proposed mechanisms were reducing inflammation, oxidative stress [20, 21, 41, 42], obesity, adipogenesis [20], HMG-CoA reductase, cholesterol absorption and intestinal transmission, apo-B100 expression [51], and lipogenic genes expression, increasing LDL receptors [52], regulating of some genes involved in lipoprotein [20] and lipid metabolism, anti-atherogenic effects, and statins-like functions [51]. Also, nesfatin can improve glucose profiles by many mechanisms [5, 54–58]. Thus, the present improvements may be attributed to increased nesfatin levels. In addition, the effect of NC on the WC can improve glucose indices [28, 55] and lipids [59, 60].

The NC in infertile men significantly increased total antioxidant capacity and reduced CRP and TNF-α levels [61]. The anti-inflammatory targets of curcumin have been mentioned TNF-α, IL-1β, NF-κB, IL-6, COX2, 5-LOX, iNOS, IL-17A, IL-17F, and IL-22 [23]. Also, several reviews have shown the anti-inflammatory effects of curcumin [27, 36, 41–49, 62–64]. Its proposed anti-inflammatory mechanisms were reducing expression and release of inflammatory factors [27, 36, 39, 44, 63, 65] and PPARγ activation [66]. Also, decreases in WC can improve inflammatory factors including TNF-α, IL-6, and hs-CRP [49, 50]. The beneficial effects of nesfatin on inflammation and oxidative stress have been reported in the previous studies including reducing the NF-κB expression, IL-6, IL-1β, TNF-α, and apoptosis [67, 68].

In only two separate studies of Jujube and *Nigella sativa* effects on nesfatin, Jujube increased liver and plasma levels of it. The reported important effects for nesfatin are appetite loss, anti-hyperglycemic, anti-inflammation, neuroendocrine regulators, reducing body fat, and metabolic regulation [69]. Thus, the improvements in glucose indices [5, 54, 56–58], lipids [57, 58], inflammation [67], and anthropometrics [5, 54, 56], and subsequently NAFLD, may be attributed to increased nesfatin levels by NC supplementation.

Some animal studies of curcumin/turmemeric supplementation have shown the beneficial effects on fatty liver and serum aminotransferases [61, 70–73]. In a study on NAFLD patients, curcumin significantly reduced fatty liver degree, ALT, and AST [17]. According to three separate reviews, curcumin can reduce ALT, AST [39, 40], ALP, GGT [40], steatosis, inflammation, and ROS [21]. However, a study of the turmeric effects on liver enzymes and fatty liver degree in NAFLD patients showed no significant changes [22]. The reason for the differences may be the type and form of supplementation. The other proposed mechanisms in improving liver enzymes and fatty liver by curcumin were reducing inflammatory markers, lipid synthesis/accumulation, anthropometric measurements [17], and oxidative stress [40, 70], PPARγ activation, and effects on glycolysis [73]. Also, the effect of nesfatin on steatosis improvement has been reported in a previous animal study [57].

The strengths were first examining the effect of NC in obese NAFLD patients, especially by assessing nefatin levels, stratified blocked randomization, the newly diagnosed NAFLD patients no receiving treatment, and assessing dietary intakes and physical activity levels. However, some limitations were no liver biopsy and measuring GGT and certain factors mentioned (PPAR-γ, etc.), self-reporting of dietary intakes and physical activity, selecting a specific center, and no checking the bioavailability and blood levels of NC.

## Conclusion

NC supplementation in overweight and obese NAFLD patients improved some markers related to obesity and NAFLD including nesfatin, QUICKI, fatty liver degree, WC, glucose indices, lipids, inflammation, and liver transaminases. The effects of NC on weight, BMI, FM, SBP, and DBP were not significant. Accordingly, the proposed mechanism for ameliorating NAFLD with NC supplementation was approved by increasing serum nesfatin levels and likely subsequently improving inflammatory, lipid and glucose profiles. Further trials on effects of curcumin are suggested, involving larger sample sizes, longer durations, non-obese patients, and considering the mentioned limitations.

## Abbreviations

ALT: Alanine transaminase
AMPK: Adenosine monophosphate-activated protein kinase
AST: Aspartate transaminase
BMI: Body mass index
CNS: Central nervous system
COX2: Cyclooxygenase-2
DBP: Diastolic blood presure
DFE: Dietary Folate Equivalent
ECL: Electrochemiluminescence
EGCG: Epigallocatechin gallate
EIA: Enzyme immunoassay
ELISA: Enzyme-linked immunosorbent assay
FBI: Fasting blood insulin
FBS: Fasting blood sugar
GGT: Gamma-glutamyltransferase
GGT: Gamma-glutamyltransferase
HbA1c: hemoglobin A1c
HDL-C: High density lipoprotein-cholesterol
HOMA-IR: Homeostasis model assessment-insulin resistance
hs-CRP: High-sensitivity C-reactive protein
IL-1β: Interleukin-1 beta
IL-6: Interleukin-6
iNOS: inducible-nitric oxide synthase
LDH: Lactate dehydrogenase
LDL-C: Low density lipoprotein-cholesterol
LXR: Liver X receptor
MAPKs: Mitogen-activated protein kinases
MCP: Monocyte chemoattractant protein
NAFLD: Non-alcoholic fatty liver disease
NF-κB: Nnuclear factor kappa B
NIOC: National Iranian Oil Company
NO: Nitric oxide
PGC-1α: PPAR-γ co-activator-1 alpha
PGC-1α: PPAR-γ co-activator-1 alpha
PPAR: Peroxisome proliferation activated receptor
QUICKI: Quantitative insulin sensitivity check index
ROS: Reactive oxygen species
SBP: Systolic blood presure
SF-IPAQ: Short Form-International physical activity questionnaire
SREBPs: Sterol regulatory element–binding proteins
SREBPs: Sterol regulatory element–binding proteins
TC: Total cholesterol
TNF-α: Ttumor necrosis factor-alpha
TPN: Total parenteral nutrition
TWRM-ANOVA: Two way repeated measures-Analysis of variance
UCPs: Uncoupling proteins
USDA: United States Department of Agriculture
